# A newborn Screening Programme for Inborn errors of metabolism in Galicia: 22 years of evaluation and follow-up

**DOI:** 10.1186/s13023-024-03204-y

**Published:** 2024-05-17

**Authors:** María L. Couce, María-Dolores Bóveda, Daisy E. Castiñeiras, María-Eugenia Vázquez-Mosquera, Sofía Barbosa-Gouveia, María-José De Castro, Agustin J. Iglesias-Rodríguez, Cristóbal Colón, José A. Cocho, Paula Sánchez

**Affiliations:** 1 Diagnosis and Treatment of Congenital Metabolic Diseases, University Clinical Hospital of Santiago de Compostela, A Coruña, Spain; 2 Health Research Institute of Santiago de Compostela (IDIS), Santiago de Compostela University, CIBERER, RICORS, MetabERN, A Coruña, Spain

**Keywords:** Age at first NBS, Dried urine samples, Intelligence quotient, Mass spectrometry, Second biomarker

## Abstract

**Background:**

There is a notable lack of harmonisation in newborn screening (NBS) programmes worldwide. The Galician programme for early detection of inborn errors of metabolism (IEM) was one of the first NBS programmes in Europe to incorporate mass spectrometry (July 2000). This programme currently screens for 26 IEMs in dried blood and urine samples collected 24–72 h after birth.

**Results:**

In its 22-year history, this programme has analysed samples from 440,723 neonates and identified 326 cases of IEM with a prevalence of 1:1351. The most prevalent IEMs were hyperphenylalaninaemia (*n* = 118), followed by medium chain acyl-CoA dehydrogenase deficiency (MCADD, *n* = 26), galactosaemia (*n* = 20), and cystinurias (*n* = 43). Sixty-one false positives and 18 conditions related to maternal pathologies were detected. Urine samples have been identified as a useful secondary sample to reduce the rate of false positives and identify new defects. There were 5 false negatives. The overall positive value was 84.23%. The fatality rate over a median of 12.1 years of follow-up was 2.76%. The intelligence quotient of patients was normal in 95.7% of cases, and school performance was largely optimal, with pedagogic special needs assistance required in < 10% of cases. Clinical onset of disease preceded diagnosis in 4% of cases. The age at which first NBS report is performed was reduced by 4 days since 2021.

**Conclusions:**

This study highlights the benefits of collecting urine samples, reduce NBS reporting time and expanding the number of IEMs included in NBS programmes.

## Introduction

Inborn errors of metabolism (IEM) constitute a growing, heterogeneous group of genetically and phenotypically complex diseases, currently comprising 1450 disorders [1]. The advent of tandem mass spectrometry (MS/MS) has allowed rapid measurement in a single sample of multiple biomarkers that serve as indicators of metabolic diseases and has led to an expansion in the number of disorders included in newborn screening (NBS) panels [2, 3]. This technique has been used to measure dozens of metabolites in the context of NBS [4], mainly to detect galactosaemia; urea cycle defects; and amino acid, organic acid, and fatty acid disorders. Some conditions, such as biotinidase (BTD) deficiency, require enzymatic activity assay [5]. The aim of NBS is to diagnose, preferably at pre-symptomatic stages, diseases that can lead to severe metabolic deficits if left untreated. Early detection, rapid diagnostic confirmation, and treatment of these hereditary pathologies can significantly alter the patient’s prognosis.

A key challenge in NBS is to minimize the number of families with healthy children who receive alarming false positive diagnoses. In metabolic diseases, increased levels of one analyte in a metabolic pathway can result in changes in the levels of other analytes or the production of secondary metabolic by-products. Relevant ratios between analytes can be calculated and used as secondary markers in order to decrease the likelihood of false positive results and thereby increase the positive predictive value (PPV) [6, 7].

The selection of diseases for inclusion in expanded NBS programmes is somewhat contentious, owing to the wide diagnostic possibilities that the new technologies and new treatments allow, and there is therefore considerable variability across European countries in the diseases that are screened for [8–10]. Few European states have legislation or laws regulating this preventive paediatric care service: screening is almost always selected on a voluntary basis (after both parents/guardians have provided written informed consent). Moreover, there is a notable lack of harmonisation of the information provided to parents about NBS programmes, which differ between countries in terms of recommendations, guidelines, and regulations [11, 12].

Long-term follow-up and evaluation of NBS programmes is essential to monitor and improve their outcomes. We first evaluated the performance of our NBS programme in Galicia (N.W. Spain) over a period of 10 years [13]. This programme is unusual in that it includes the simultaneous collection of both blood and urine samples from all newborns. Here, we present the findings of theevaluation of the Galician NBS programme over a 22-year period, and reflect upon the experience accumulated over this time, as well as the advances made in our understanding of the natural history of these diseases.

## Patients and methods

All Galician newborns with abnormal NBS results suggestive of IEM are referred for evaluation to the Unit of Diagnosis and Treatment of Congenital Metabolic Diseases (“UDyTEMC”) of the Clinical University Hospital of Santiago de Compostela, a national reference centre (CSUR) for IEM. The present study includes all newborns referred from the NBS programme between July 1, 2000 and December 31, 2022.

### Newborn screening

Since August 2020, the NBS programme in Galicia has collected a dried blood spot sample and a urine sample (on Whatman 903 paper) from all participating newborns 24–72 h after birth, after at least 24 h of milk intake. In the majority of cases samples are collected in the maternity ward, prior to hospital discharge. Prior to August 2020, samples were collected either between 48 and 72 h after delivery (from January 2003 to July 2020) or between 5 and 8 days of age (from July 2000 until December 2002). For the purposes of the present study, the age at sample collection, receipt, and laboratory report was determined. Urine samples were collected by placing a paper slip between the diaper and the dry genitals (free of creams, talcum powder, etc.). Once impregnated with urine, the paper was dried at room temperature, taking care to avoid contamination by faeces. All the dried blood spot (DBS) specimen handling, packaging, and transport are performed in accordance with CLSI recommendations [14]. DBS specimens are shipped using a basic triple-packaging system.

Levels of amino acids, acylcarnitines, and hexoses monophosphate in blood samples were determined by MS/MS using an Applied Biosystems Sciex API 4000 apparatus [15, 16], and biotinidase (BTD) activity by colorimetric assay [5]. Urine samples were analysed for cystine using the de Brand test and for galactose using thin layer chromatography [17]. In cases of a positive result, urine samples underwent repeated or additional analyses using second-tier tests: MS/MS for amino acids, acylcarnitines, organic acids, and acylglycines [18, 19]. All newborns were screened for a panel of metabolic disorders including amino acid disorders [phenylketonuria (PKU)/mild hyperphenylalaninaemia (HPA), classic homocystinuria (HCU), tyrosinaemia type 1 (TYR1), maple syrup urine disease (MSUD)]; urea cycle defects [citrullinaemia type 1, argininosuccinic aciduria, argininaemia]; organic acid disorders [propionic acidaemia (PA), methylmalonic acidaemias (MMA/Cbl), isovaleric acidemia (IVA), glutaric aciduria type 1 (GA-1), 3-hydroxy-3-methyl glutaric aciduria (HMG), 3-methylcrotonyl-CoA carboxylase deficiency (3-MCCD), ß-ketothiolase (BKT) deficiency, 3-methylglutaconic aciduria (3-MGA)]; fatty acid disorders [medium chain acyl-CoA dehydrogenase deficiency (MCADD), very long-chain acyl-CoA dehydrogenase deficiency (VLCADD), long-chain 3-hydroxyacyl-CoA dehydrogenase deficiency (LCHADD), carnitine-acylcarnitine translocase deficiency (CACTD), carnitine transporter defect (CTD), carnitine palmitoyltransferase (CPT) deficiencies, multiple acyl-CoA dehydrogenase deficiency (MADD)]; galactosaemias [galactose-1 phosphate uridyltransferase (GALT) deficiency, galactokinase (GALK) deficiency]; BTD deficiency; and cystinurias. The NBS programme in Galicia screens for 26 metabolic diseases as primary diagnosis. Initially, hypermethioninaemia due to methionine adenosyl transferase (MAT I-III) deficiency and short-chain acyl-CoA dehydrogenase deficiency (SCADD) were also included. But the greater knowledge with the evolution has led to their not being included as a primary diagnosis since 2010.

Percentiles of MS/MS-measured analyte concentrations in blood samples from healthy and diseased newborns were periodically reported to the Region 4 Genetics Collaborative Project (currently known as CLIR) [20]. If the results of the analyses were clearly aberrant and suggestive of a severe disorder, the patient was immediately referred to UDyTEMC. If the results lay outside the reference range but were not so aberrant as to constitute clear proof of severe disease, a second sample was requested by the NBS laboratory. If this second sample also tested positive, the patient was referred to the clinical unit.

In all cases with biochemical markers suggestive of IEM, a genetic/molecular study was performed for confirmation.

### Clinical outcomes of screened individuals with an IEM diagnosis

In the present study the following patient characteristics were evaluated to determine the status of each patient at diagnosis: presence or absence of clinical symptoms; biochemical markers suggesting diagnosis; diagnosis; diagnostic confirmation methods; and use of dialysis techniques or other detoxification measures. The following variables measured during follow-up examinations were included: age; use of dietary and/or pharmacological treatment; follow-up period; and outcome (including evolving clinical symptoms, metabolic decompensations, and death). Metabolic decompensation was defined as any event requiring hospitalization due to subclinical biochemical derangement or any clinical presentation indicating clinical decompensation. Onset of first metabolic decompensation in the neonatal period (30 days of life) was classified as early onset (EO), and onset at a later age as late onset (LO).

Cases in which biochemical markers were abnormal at NBS but normal upon diagnostic testing were considered false positives. Cases in which biochemical markers remained altered at the first post-NBS evaluation but later normalized spontaneously were considered transitory. Because our unit cares for the vast majority of patients with metabolic diseases in Galicia, cases in which a patient was not detected by screening but was later diagnosed with any of the IEMs included in the NBS programme were considered false negatives.

The psychomotor development index (PDI) or intellectual quotient (IQ) of survivors were evaluated using the Brunet Lézine Scale for infants, the McCarthy Scales of Children’s Abilities (MSCA) for preschool children, and the revised Wechsler Intelligence Scale for Children (WISC-R) for children over the age of 6 years. PDIs and IQs > 85 were considered normal. This study was approved by our hospital‘s ethics committee.

### Statistical analysis

A descriptive statistical analysis of the study population was performed, analysing parameters of centralization (mean and median) and dispersion (maximum and minimum, and range) and percentiles. Sensitivity, specificity, and positive and negative predictive values were also evaluated.

## Results

In Galicia, the MS/MS NBS programme began on July 1, 2000. From its inception up to December 31, 2022 (22.5 years) 440,723 neonates were analysed. The annual coverage of neonates screened was 98–99% between 2000 and 2008, and 100% from 2009 to the present. Since January 2003, when sampling between 24 and 48 and 72 h of life was established and samples were collected from the majority of newborns at their corresponding Health Centre and sent by parents by post, the average age of collection of the first NBS sample was 72 h after birth, the age (percentile [P]-50) at sample reception at the laboratory was 6 days, and the age (P50) at first NBS report was at 10 days. Since 2022, samples have arrived at the laboratory for analysis faster owing to implementation of direct transport of NBS samples from all Galician hospitals to the NBS laboratory. The age (P50) at first NBS report (suspected diagnosis) was 5–7 days (Table 1).

**Table 1:** Follow-up from sample collection to the result of the Metabolic Laboratory

	Years 2003–2020		Years 2021–2022	
	P50	P95	P50	P95
Time (1) Age at first NBS sample collection	3 days	5 days	2 days	4 days
Time (2) Age at sample receipt at laboratory	7 days	15 days	4 days	11 days
Time (3) Age at first NBS report (suspected diagnosis)	9 days	18 days	5 days unless organic acid evaluation was required, in which case the P50 was 7 days	15 days

NBS, newborn screening; *P*, percentile

During the study period, 326 cases of IEM were identified (1 per 1351 newborns), and were followed for a median duration of 12.1 years. During the first 10 years of the programme, as previously reported [13], 137 cases were identified in a population of 210,165 newborns, increasing to 189 cases over the following 12 years (population of 230,558). As shown in Table 2, the most frequently detected IEMs were hyperphenylalaninaemias (118 cases; mild HPA, 1/5650; PKU, 1/11,018); MCADD (26 cases; 1/16,951); galactosaemias (20 cases; GALT deficiency, 1/34,024; GALK deficiency, 1/62,960) and cystinurias (43; 1/10,249) (Table 2). Urinary biochemical markers were of significant value in enabling positive detection (GALK, cystinurias) or complementing positive detection of blood tests (e.g. assessment of organic acids or acylglycines in the case of certain diseases). Diagnoses were confirmed by molecular studies.

**Table 2:** Characteristics of 326 cases diagnosed by NBS (total newborns screened: 440,723)

Disorders	No. Subjects	Prevalence (live births)	Abnormal biochemical markers in the screening test (median [range])	Dietary/Pharmacologic treatment	Dialysis therapy	Mean follow-up (months)	MD EO	MD LO	Mean PDI/IQ	Current status
***Amino acid disorders***										
Phenylketonuria	40	1/11,018	Phe (b) 602 µM (188–1418) Phe/Tyr: 10.5 (3.6–29.5)	Yes	No	12 y 5 m	0	0	104	Normal life with treatment. Appropriate LE
Mild HPA	78	1/5650	Phe (b): 170 µM (102–393) Phe/Tyr: 2.7 (1.2–7.2)	No	No	11 y 8 m	0	0	110	Normal life Appropriate LE
Classic HCY	1	1/440,723	Met (b): 59 µM Hcy (u): 22 mmol/mol crea	Yes	No	18 y 4 m	0	0	133	Normal life with treatment. Appropriate LE
TYR I	3	1/146,908	Tyr (b): 662 µM (101–729) Suac (b): 15.4 µM	Yes	No	13 y 6 m	1 case	0	86	Normal life with treatment. Appropriate LE ADHD (1 case)
MSUD	10	1/44,072	XLeu (Leu + Ile) (b): 1368 µM (514–3367) Val (b): 644 µM (244–925)	Yes	4 cases	12 y 9 m	6 cases	6 times/4 cases	113	2 exitus not related with their metabolic disease. Appropriate LE
Citrullinaemia type 1	3	1/146,908	Cit (b): 904 µM (139–1096) Orotic acid (u): 80 mmol/mol crea (4.2–1153)	Yes	2 cases	12 y 9 m	2 cases	1	79	1 exitus due to sepsis during neonatal onset. Appropriate LE
Argininosuccinic aciduria	2	1/220,362	Cit (b): 19.5–62 µM ASA (u): 186–1785 mmol/mol crea	Yes	No	9 y 8 m	0	0	95	Normal life with treatment. ADHD(1 case)
Argininaemia	1	1/440,723	Arg (b): 35 µM Orotic acid (u): 639 mmol/mol crea	Yes	No	16 y 2 m	0	0	90	Normal life with treatment. Appropriate LE
*Secondary conditions*:										
MAT I/III deficiency	15	1/29,381	Met (b): 82 µM (45.1–147)	Partial	No	15 y 5 m	0	0	> 95	Normal life. Appropriate LE
Alcaptonuria	4	1/110,181	Homogentisic acid (u): 245 mmol/mol crea (190–7000)	Yes	No	12 y 7 m	0	0	> 95	Normal life with treatment. Appropriate LE
TYR III	1	1/440,723	Tyr (b): 392 µM; Suac (b): 0.58 µM (normal)	Yes-	No	9 y	0	0	124	Normal life with treatment. Appropriate LE
***Organic acid disorders***										
PA	3	1/146,908	C3 (b): 8.42 µM (8.2–14.6) C3/C2: 1.08 µM (0.51–1.7) C3/C16: 3.24 µM (2.8–7.4)	Yes	2 cases	7 m	3 cases	2	NA	Exitus at 2 m, 4 m and 12 m of life
MMA	3	1/146,908	C3 (b): 5.2–5.8 µM C3/C2: 0.49–0.68 µM MMA (u): 241–874 mmol/mol crea	Yes	1 case	8 y 11 m	1 case	0	113	Normal life with treatment. Appropriate LE
IVA	1	1/230,998	C5 (b): 3.43 µM; C5/C2 (b): 0.26	Yes	No	9 y 8 m	0	0	110	Normal life with treatment. Appropriate LE
MMA CblC, CblD	4	1/110,181	C3 (b): 7.73 µM (2.83–152) MMA (u): 887 mmol/mol crea (89–2274) Hcy (u): 30 mmol/mol crea (ND–86.9)	Yes	No	10 y 8 m	0	0	78	Normal life with treatment (2) Retinopathy and developmental delay: 2 cases. 2 cases with special needs in school
GA-1	7	1/62,960	Glutarylcarnitine (b): 1.62 µM (0.27–4.23) Glutarylcarnitine (u): 52.9 mmol/mol crea (6.5–132)	Yes	No	16 y 8 m	0	1	95	Normal life with treatment: 5/6 Slight cognitive delay: 1/6 1 with special needs in school + speech therapy
HMG	2	1/220,362	C5OH (b): 0.93–1.88 µM 3-methylglutarylcarnitine (b): 0.54–0.57 µM	Yes	No	13 y 5 m	0	1	98	Normal life with treatment. Appropriate LE
3-MCCD	10	1/44,072	C5OH (b): 1.35 (0.74–5.3) 3-OH isovaleric acid (u): 318 mmol/mol crea (60–3521)	Yes	No	12 y 10 m	0	0	110	Normal life with treatment Appropriate LE
*Secondary conditions*:										
Combined malonic and methylmalonic aciduria	4	1/110,181	C3 (b): 1.16 µM (0.96–3.1) MMA (u): 845 mmol/mol crea (772–948)	No	No	5 y 11 m	0	0	97	Normal life. Appropriate LE
Disorders of glutathione metabolism: GSSD	1	1/440,723		Yes	No	19 y	1	42	55	Developmental delay, severe hypoacusis and frequent hemolytic crisis. Special needs in school
***Fatty acid β-oxidation disorders***										
MCADD	26	1/16,951	C8 (b): 9.5 µM (0.42–15.8) C8/C10 (b): 5.3 µM (0.09–13.71)	Yes	No	10 y 7 m	0	1	> 95	Exitus (1) during respiratory infection Normal life with treatment (25/26)
LCHADD	2	1/220,362	C16OH (b): 0.79–0.68 µM; C18: 1OH(b): 0.97–0.51 µM; C18OH (b): 1.4–0.7 µM	Yes	No	18 y 3 m	0	8	> 95	Normal life with treatment Retinopathy: 1 case Appropriate LE
VLCADD	3	1/146,908	C14 (b): 0.98 µM; C14: 1 (b): 0.58 µM	Yes	No	9 y 3 m	0	0	> 95	Normal life with treatment Appropriate LE
CACTD	1	1/440.723	C16 (b): 16.4 µM; C18 (b): 2.83 µM; C18: 1 (b): 4.0 µM	Yes	No	9 m	1	1	85	Exitus due to a arrhythmia at 9 m during infection
CTD	2	1/220,362	C0 (b): 2.3–9.8 µM	Yes	No	13 y 1 m	0	0	> 95	Normal life with treatment Appropriate LE
MADD	1	1/440,723	C8 (b): 0.44 µM; C10 (b): 0.806 µM; C5DC (b): 0.22 µM	Yes	No	1 y 11 m	1	0	> 95	Normal life with treatment Appropriate LE
CPT2 deficiency	1	1/440,723	C16 (b): 12.52 µM; C16: 1 (b): 1.02 µM; C18 (b): 5.53 µM	Yes	No	3 y 2 m	0	0	> 95	Normal life with treatment Appropriate LE
SCADD	12	1/36,726	C4 (b): 1.21 µM (0.35–2.16) EMA (u): 31.7mmo/mol crea (16.5–102)	only recommendation to avoid fasting	No	11 y 6 m	0	0	> 95	Normal life. Appropriate LE
***Galactosaemias***										
GALT deficiency	13	1/34,024	Gal-1-P (b): 1.61 mM (0.41–3.99)	Yes	No	10 y	1 case	0	90	Normal life with treatment: 11 cases Developmental delay: 2 cases with special needs in school Ovarian insufficiency: 2 cases.
GALK deficiency	7	1/62,960	Gal-1-P (b): Normal, Gal (u) elevated	Yes	No	10 y 4 m	0	0	> 95	Normal life with treatment Appropriate LE
*Secondary conditions*:										
GALE deficiency	7	1/62,960	Gal-1-P (b): 2.59 mM (0.95–4)	No	No	13 y 4 m	0	0	> 95	Normal life. Appropriate LE
GALM deficiency	2	1/220,362	Gal-1-P (b): Normal, Gal (u) elevated	Yes	No	8 y 2 m	0	0	> 95	Normal life with treatment Appropriate LE
***Disorders of biotin metabolism***										
BTD partial deficiency	9	1/48,969	BTD activity (b): 21.5% (13.6–25)	Yes	No	13 y	0	0	> 100	Normal life with treatment Appropriate LE
BTD total deficiency	4	1/110,181	BTD activity (b): 1.3% (0–3.1)	Yes	No	13 y 10 m	0	0	> 100	Normal life with treatment Appropriate LE
***Amino acid transport defects***										
Cystinuria	43	1/10,249	Cys (u): 383 mg/g crea (173–1614)	Yes	No	14 y 5 m	0	0	> 100	Normal life with treatment Urolithiasis: 1 case

ADHD: Attention Deficit Hyperactivity Disorder; Arg, arginine; ASA, argininosuccinic acid; b, blood; BTD, biotinidase ; C0, free carnitine; C2, acetyl carnitine; C3, propionyl carnitine; C4, butyryl-carnitine; C5, isovaleryl carnitine; C5OH, 3-hydroxyisovalerilcarnitine; C5DC, glutarylcarnitine; C8, octanoylcarnitine; C10, decanoylcarnitine ; C16, palmitoylcarnitine; CACTD, carnitine-acylcarnitine translocase deficiency; CblC, cobalamine C; CblD, cobalamine D; Cit, citrulline; CPT2, carnitine palmitoyltransferase II deficiency; crea: creatinine; CTD, carnitine transporter deficiency; Cys, cysteine; EMA, ethylmalonic acid; EO, early onset; GA-1, glutaric aciduria type 1; Gal, galactose; Gal-1-P, galactose-1-phosphate; GALE, galactose epimerase; GALK, galactokinase; GALM, galactose mutarotase; GALT, galactose-1-phospahte uridyltransferase; GSSD, glutathione synthetase deficiency; Hcy, homocysteine; classic HCY, classic homocystinuria; HMG, 3-hydroxy-3-methylglutaric aciduria; HPA, hyperphenylalaninaemia; Ile, isoleucine; IVA, isovaleric acidaemia; LCHADD, long chain 3-hydroxyacyl-CoA dehydrogenase deficiency; Leu, leucine; LO, late onset (after the neonatal period); MADD, multiple acyl-CoA dehydrogenase deficiency; MAT I/III deficiency, methionine adenosyltransferase I/III deficiency; MCADD, medium-chain acyl-CoA dehydrogenase deficiency; 3-MCCD, 3-methylcrotonyl-CoA carboxylase deficiency; MD, metabolic decompensation; Met, methionine; MMA, methylmalonic acidaemia; m, months; MSUD, maple syrup urine disease; ND, not detectable; PA, propionic acidemia; PDI/IQ, psychomotor developmental index/ intelligence quotient; Phe, phenylalanine; SCADD, short-chain acyl-CoA dehydrogenase deficiency; Suac, succinylacetone; Tyr, tyrosine; TYR I, tyrosinaemia type I; TYR III, tyrosinaemia type III; u, urine; Val, valine; VLCADD, very long-chain acyl-CoA dehydrogenase deficiency; y, years

Clinical signs of intoxication were already evident at a median age of 3 days of age in 13 patients (4%) before NBS results were available, including 2 cases of citrullinemia type 1, 6 cases of MSUD, 3 cases of PA, and 1 case each of MMA mutase deficiency and CACTD (Table 3). Nine of these cases (2 with citrullinemia type 1; 4 with MSUD; 2 with PA; and 1 with MMA) required extracorporeal removal therapy. Seven of these cases occurred before 2003, when samples were collected later (at 5–8 days of age).

**Table 3:** Cases with clinical onset prior to NBS results (period 2000–2022)

Case	Age at detection by NBS (days)	Age at clinical onset (days)
1. Citrullinaemia type 1	9	2
2. Citrullinaemia type 1	8	3
3. MSUD	5	4
4. MSUD	7	5
5. MSUD	8	4
6. MSUD	10	3
7. MSUD	6	3
8. MSUD	9	4
9. Propionic acidaemia	8	2
10. Propionic acidaemia	7	3
11. Propionic acidaemia	7	3
12. MMA mutase deficiency	8	3
13. CACTD	6	1

CACTD, carnitine acylcarnitine translocase deficiency; MMA, Methylmalonic acidaemia; MSUD: maple syrup urine disease; NBS: newborn screening

Higher frequencies of LO metabolic decompensation were observed for MSUD, organic acidurias, and urea cycle defects. Four patients with MSUD presented evolutionary episodes of mild-to-moderate metabolic decompensation in the context of infectious episodes. All responded well to emergency regime early onset, except in one case of severe LO decompensation in a patient with cerebral oedema and intracranial hypertension, who required haemofiltration in the context of an adenovirus infection with slowly reversible neurological sequelae.

Of the 326 cases of IEM detected by screening whose care was centralized in our Unit, 9 patients died. Four of those patients have been previously described by our group (13), and correspond to 3 patients with organic aciduria (severe forms with neonatal onset) who died due to infectious conditions at 2, 4, and 12 months of life respectively, and 1 patient with asymptomatic MCADD, who died with acute respiratory disease due to metabolic decompensation. From 2010, 5 patients died: 1 citrullinaemia patient who died during the neonatal period due to sepsis; 1 girl with PA who died at 8 months of age due to septic shock; 1 CACTD patient who died due to fatal ventricular fibrillation during an adenovirus and enterovirus coinfection at 9 months of age; and 2 MSUD patients who died at 6 and 20 years of age due to causes unrelated with their metabolic disease. The fatality rate for our study population was 2.76%.

All patients survivors of β-oxidation disorders, organic acidurias (including biotinidase deficiency), urea cycle disorders and MSUD, GA 1, TYR 1, were asymptomatic, with the exception of 1 case of LCHAD with retinopathy; 2 cases of CblC with poor visual acuity, nystagmus, and developmental delay; and 2 cases of galactosaemia with ovarian failure and mild disability despite good metabolic control. The IQ was normal in 95.7% of cases. School success was very good, with special needs assistance required in < 10% of cases.

As shown in Table 4, there were 79 confirmed false positives (0.017%) during the study period. The biochemical marker for which elevated values were most frequently non-pathological was citrulline (Cit), with 20 false positives: citrullinaemia 1 was ruled out by tests that included determination of ammonium and amino acids in plasma and urine, and urinary organic acids (including orotic acid), and no instances of citrin deficiency were detected. This was followed by BTD activity (b), with 11 false positives. Enzymatic activity is measured by colorimetric assay using biotinyl-p-aminobenzoate as a substrate. There were 10 cases of transient elevations in urine levels of methylmalonic acid (mean, 113 mmol/mol creatinine) due to maternal B_12_ deficiency; 4 cases of elevated 3-hydroxyisovalerilcarnitine (C5OH) due to placental transfer of maternal metabolites (leading to diagnoses of previously undetected 3-MCCD in the mothers); and 4 cases of free carnitine (C0) < 6 µM leading to diagnosis of maternal CTD.

**Table 4:** Characteristics of the 61 false positive cases and 18 maternal defects (period 2000–2022)

No. of cases	Possible unconfirmed entity	Abnormal biochemical marker	Level	Cut-off ª
3	Mild HPA	Phe (b) Phe/Tyr (b)	150.8 µM [143–155] 1.62 [1.63–1.96]	120 µM 1.96
4	IVA	C5 (b)	2.57 µM [0.81–7.39]	0.46µM
3	GALT deficiency	Gal-1-P (b) with galactose (u) normal	1.49 mM [0.81–2.4]	0.7 mM
6	GALK deficiency	Galactose (u) with Gal-1-P(b) normal	elevated	Qualitative findings
20	Citrullinaemia	Cit (b)	106 µM [36–265]	31µM
5	MCADD (3 of them carriers)	C8 (b)	0.62 µM [0.34–0.75]	0.26 µM
1	VLCADD	C14 (b) C14:1 (b)	0.87 µM 0.53 µM	0.52 µM 0.47 µM
6	CPT1	C0 (b) C16 (b) C18 (b)	73.1 µM [56.9–91.1] 0.83 µM [0.45–1.5] 0.58 µM [0.4–0.84]	9.5–75 µM 0.41–7.1 µM 0.24–2 µM
2	SCADD	C4 (b)	1.11 µM [0.94–1.29]	0.94µM
11	BTD deficiency	Biotinidase activity (b)	0.12 Aus [0.09–0.16]	0.20 Aus
**Transitory alterations due to undiagnosed maternal IEM or vitamin deficiencies**				
4	CTD	C0 (b)	5.3 µM [4.9–5.6]	9.5 µM
4	3-MCCD	C5OH (b)	3.3 µM [2.94–3.69]	0.35 µM
10	MMA	Methylmalonic acid (u)	113 mmol/mol crea	13 mmol/mol crea

ª As of June 2010, cut-offs were re-evaluated every 6 months; Aus, absorbance units; BTD, biotinidase deficiency; C0, free carnitine; C2, acetylcarnitine; C3, propionylcarnitine; C4, butyrylcarnitine; C5, isovalerylcarnitine; C5OH, 3-hidroxyisovalerilcarnitine; C8, octanoylcarnitine; C10, decanoylcarnitine ; C16, hexadecanoyl-L-carnitine; Cit, citrulline; CPT1, carnitine palmitoyltransferase I deficiency; CTD, carnitine transporter deficiency; Gal-1-P, galactose-1-phosphate; GALK, galactokinase; GALT, galactose-1-P uridyltransferase; HPA, hyperphenylalaninaemia; IVA, isovaleric aciduria; MCADD, medium chain acyl-CoA dehydrogenase deficiency; 3-MCCD, 3-methylcrotonyl-CoA carboxylase deficiency; MMA, methylmalonic acidaemia; Phe, phenylalanine; SCADD, short-chain acyl-CoA dehydrogenase deficiency; Tyr, tyrosine; U, urine; VLCADD, very long-chain acyl-CoA dehydrogenase deficiency

Of the 5 patients with false negative screening results, 1 patient in 2002 with TYR 1 had a tyrosine (Tyr) concentration of 162 µM at screening (cutoff, 175 µM) and the first-level succinylacetone marker was not yet implemented. This patient was identified following acute liver failure at age 1 month. Another was a low excreting GA-1 patient with a blood glutarylcarnitine concentration of 0.13 µM at screening (99.9 percentile 0.18 µM). In this case, clinical onset occurred with acute encephalopathy at age 8 months, and retrospective determination of glutarylcarnitine in the urine screening sample revealed a concentration of 18.2 mmol/mol creatinine (99.9 percentile, 2.48 mmol/mol creatinine). A third patient, who had MADD, was admitted to hospital at age 13 days with cardiomyopathy and acute metabolic acidosis. The fourth patient was diagnosed with benign HPA at age 6 years after a newborn sister screened positive for this IEM. At this stage, the older sibling‘s phenylalanine (Phe) concentration was 315 µM (Phe/Tyr ratio 4.2), whereas the Phe concentration observed at screening was 141 µM (Phe cutoff at time of screening, 150 µM) and the Phe/Tyr ratio 1.6 (cutoff at time of screening, 2.0). The fifth case, the most recent, was a patient with PA with onset of clinical symptoms at 18 months of life (mild metabolic decompensation during an infection). The results of NBS sample analysis at 3 days of age were as follows: C3 (b), 8.6 µM; C3/C2 (b), 0.5 µM; C3/C16 (b), 3.7 µM; propionylglycine (u), 6.3mmol/mol creatinine and was considered normal screening.

The overall PPV of the programme was 84.23% and the negative predictive value 99.99% (we assume the latter as the maximum value; hypothetical undiagnosed situations of which we are not aware could imply a lower value), with a sensitivity of 98.49%98.49% and a specificity of 99.98%.

## Discussion

The Galician Neonatal Screening Programme was one of the first European programs to incorporate MS/MS technology into neonatal screening, and pioneered this approach in Spain. Over the last 22 years this screening programme has identified 547 cases patients with endocrine, metabolic, haematological, respiratory-digestive based pathologies, 326 with IEMs. Overall sensitivity during this period has been higher than that achieved during the programme’s first 10 years (98.49% vs. 97.16%) [13], while specificity has remained unchanged (99.98%). The prevalence of IEMs detected in the Galician programme, which is one of the largest such programmes in Europe, is 1 case per 1,351 newborns. This prevalence is higher than those reported in other European programmes (Germany, 1:2,920 [21]; Portugal, 1:2,396 [22]; Denmark, Greenland and the Faroe Islands, 1:4,942 [23], and in other Spanish regions such as Murcia (1:1,884) [24]. Although second tier blood tests have improved and increased their knowledge in recent years [4], urine also continues to be an excellent source of second-level markers [25], reducing the number of false positives by 0.017% and helping to exclude cases of transitory alterations due to IEM in mother or maternal vitamin deficiencies. Urine analysis also enables detection of entities that are not biochemically expressed in the blood, such as cystinuria and GALK. The higher number of disorders screened and the inclusion of urine sample may explain the higher number of positive screening results in our region. The frequency of one of the most common false positives, elevated citrulline, has declined in recent years since the introduction of tests for orotic and argininosuccinic acids at the confirmatory analysis stage.

The most frequent IEMs in the Galician programme include hyperphenyalalaninemias, cystinuria, and MCADD. Overall, the most frequent groups of detected entities were amino acid disorders (79/326) followed by fatty acid β-oxidation disorders (46/326); organic acidurias (36/326); and galactosaemias (20/326).

A positive diagnosis by NBS implies a marked difference in prognosis, as evidenced by the high rate of symptom-free survival in our patients and the low case fatality rate (2.76% for IEMs): this figure is comparable to the 3.1% (7/219) reported by Frazier et al. among patients followed up for 8 years after diagnosis of disorders detected by MS/MS-based NBS [26], and dramatically lower than that reported in series of patients with organic acidurias and β-oxidation disorders diagnosed through clinical symptoms (greater than 30%) [27, 28]. However, NBS cannot prevent all the disease manifestations in screened individuals, highlighting the need for safer and more effective therapies. Among patients screened by our programme, despite accurate diagnosis and good adherence to the appropriate treatment, 2 females with classical galactosaemia developed ovaric insufficiency, 1 patient with LCHADD retinopathy and 2 out of 3 patients with MMA CblC developed retinopathy and nystagmus with poor visual acuity.

Despite reductions over the course of the program’s existence in the time taken to obtain results, clinical onset of certain IEMs continues to precede diagnosis in a minority of cases (4%). The earliest onset of an IEM among our patients corresponded to a patient with CACTD who developed severe hypoglycaemia and metabolic acidosis 20 h after birth. Any neonatal decompensations occurred after the positive NBS result had been reported, underlining the importance of obtaining early NBS results. Indeed, the risk of neonatal decompensation increases with each additional day by which results are delayed [29]. As described here, this is particularly important in patients with urea cycle disorders, PA, MMA, MSUD, and CACTD. While optimization of screening is a complex process, the age (P50) at first NBS report (i.e. suspected diagnosis) was reduced by 4 days since 2021. This was achieved by introducing changes at 3 key stages of the process: age at first NBS sample collection; age at sample receipt at laboratory; and age at first NBS report. We identified 2 key parameters where improvement is essential. The first is sample quality. Workshops will be required to provide training to health personnel responsible for sample collection in order to reduce the number of invalid samples received, thereby reducing delays in sample processing and treatment initiation. The second is the speed in transporting the samples to the laboratory, whose efficiency improved greatly from 2022.

This study has several limitations that should be noted. The first is the potential identification of individuals with attenuated disease variants, which could magnify the programme results. The second is the inclusion of entities such as SCADD and MAT I-III given that, although the identification of these pathologies was considered a primary objective when the program began, both were removed from the NBS panel in line with advances in knowledge. Finally, general understanding of disease markers and the availability of effective treatments has changed over the course of the years of screening program. Nonetheless, our data describe the detection and follow-up of a broad range of IEMs over more than 2 decades, in contrast with the majority of other published series, in which the time of evolution is generally shorter [21, 30].

The future of neonatal screening is currently at a crossroads, with much debate as to which diseases should be incorporated into new screening panels [31–35], how screening programs can be harmonized [8, 36, 37], the possible incorporation of -omics techniques [38, 39], and the ethical, legal, and social ramifications of incorporating genetic screening methods using massive genome sequencing [40–44]. This underscores the need to establish surveillance protocols, registries of results of NBS, and quality assessment processes, together with the evaluation of potential new diseases susceptible to be include in NBS.

## Conclusions

Our data derived from Galicia’s expanded NBS programme, which has been running for 22 years, demonstrates that early diagnosis and timely initiation of therapy result in a favourable clinical evolution in the majority of patients with IEMs. Optimization of the programme and the inclusion of urine sample analysis has resulted in a very low number of false positives. Clinical onset of disease precedes NBS screening results in 4% of cases, emphasizing the need for faster screening turnaround times to ensure earlier disease detection. Finally, advances in knowledge of IEMs should be matched by the inclusion of additional entities in NBS programs.

## Data Availability

The data is available on a restricted basis to authorized physicians of the Metabolic Unit.

## Abbreviations

BTD: Biotinidase deficiency
BKT: ß-ketothiolase deficiency
CACTD: Carnitine-acylcarnitine translocase deficiency
CPT: Carnitine palmitoyltransferase
CTD: Carnitine transporter defect
DBS: Dried blood spot
EO: Early onset
GA-1: Glutaric aciduria type 1 HCU classic homocystinuria
GALK: Galactokinase (GALK)
GALT: Galactose-1 phosphate uridyltransferase
HMG: 3-hydroxy-3-methyl glutaric aciduria
HPA: Mild hyperphenylalaninaemiaIEM inborn errors of metabolism
IVA: Isovaleric acidemia (IVA)
LCHADD: Long-chain 3-hydroxyacyl-CoA dehydrogenase deficiency
LO: Late onset
MADD: Multiple acyl-CoA dehydrogenase deficiency
MAT: I-III methionine adenosyl transferase
MCADD: Medium chain acyl-CoA dehydrogenase deficiency
MMA: Methylmalonic acidaemias
MS/MS: Tandem mass spectrometry
MSUD: Maple syrup urine disease
NBS: Newborn screening
PA: Propionic acidaemia
PKU: Phenylketonuria
PPV: Positive predictive value
SCADD: Short-chain acyl-CoA dehydrogenase deficiency
TYR1: Tyrosinaemia type 1
UDyTEMC: Unit of Diagnosis and Treatment of Congenital Metabolic Diseases
VLCADD: Very long-chain acyl-CoA dehydrogenase
3-MCCD: 3-methylcrotonyl-CoA carboxylase deficiency
3-MGA: 3-methylglutaconic aciduria
